# Feasibility of referral to a therapist for assessment of psychiatric problems in primary care – an interview study

**DOI:** 10.1186/s12875-019-1007-7

**Published:** 2019-08-19

**Authors:** Agneta Pettersson, Sonja Modin, Henna Hasson, Ingvar Krakau

**Affiliations:** 1 Procome, Medical Management Centre, Department of Learning, Informatics, Management and Ethics, Karolinska Institutet, Tomtebodavägen 18 A, SE-171 77 Stockholm, Sweden; 2 Swedish Agency for Health Technology Assessment and Social Assessment, SE-102 33 Stockholm, Sweden; 3 Division of Family Medicine and Primary Care, Department of Neurobiology, Care Sciences and Society, Karolinska Institutet, Alfred Nobels allé 23 D2, SE-141 83 Huddinge, Sweden; 4 Centre for Epidemiology and Community Medicine, Stockholm County Council, Stockholm, Sweden; 5 Department of Medicine Solna, Karolinska Institutet, Clinical Epidemiology Unit T2, Karolinska University Hospital, SE-171 76 Stockholm, Sweden

**Keywords:** Depression, Primary care, Qualitative content analysis, Teamwork, Barriers and facilitators, Feasibility, COM-B

## Abstract

**Background:**

Depression and anxiety disorders are common in primary care. Comorbidities are frequent, and the diagnoses can be difficult. The Mini-International Neuropsychiatric Interview (MINI) can be a support in the clinical examination of patients with complex problems. However, for family practitioners (FPs), time and perceptions about structured interviews can be barriers to the MINI. An inter-professional teamwork process where FPs refer a patient to a therapist for a MINI assessment represents one way in which to address the problem. The results are fed back to the FPs for diagnosis and treatment decisions.

The purposes of this study were to explore if the process was feasible for FPs, patients and therapists in Swedish primary care, and to identify factors influencing the process, using the COM-B model.

**Methods:**

FPs at two primary care centers (PHCC) in Stockholm were offered the opportunity to refer patients to in-house therapists. Semi-structured interviews or focus groups were conducted with 22 patients, 17 FPs and three therapists to capture their experiences and perceptions. Inductive content analysis for each group of participants was followed by triangulation across groups. Finally, the categories obtained were fitted to the components in the COM-B.

**Results:**

Therapists at both PHCCs conducted the MINI. The intended process was adopted at one PHCC. At the second PHCC, the responsibilities for the diagnosis and treatment of patients referred were transferred to the therapist. The patients were satisfied, as they appreciated multi-professional examinations. The FPs’ competence in psychiatry, actual access to therapists, beliefs that the referrals saved the FPs time and effort, and established habits influenced whether patients were referred. Existing routines and professional expectations for work content influenced the degree of cooperation between the therapists and the FPs.

**Conclusions:**

An inter-professional diagnostic process where FPs refer patients to a therapist for assessment and the results are fed back to the FPs can be feasible. Feasibility depends on access to a therapist, the perceptions of roles and competences among FPs and therapists, and strategies for supporting teamwork.

**Electronic supplementary material:**

The online version of this article (10.1186/s12875-019-1007-7) contains supplementary material, which is available to authorized users.

## Background

Psychiatric problems, such as depression, anxiety and stress-related disorders, are common in the population [1–4], and most care seekers with these problems are managed in primary care [5]*.* One out of four patients in primary care are reported to suffer from depression or anxiety [6, 7]. However, it has been questioned whether primary care has sufficient prerequisites for the management of mental problems [5, 8, 9]. Issues related to structural limitations and professionals’ competence in mental health have been raised. This article deals with a central aspect of the management of psychiatric problems, the diagnostic process. A formal diagnosis setting is important, as the recording of a diagnosis is associated with a higher chance of adequate treatment [10, 11].

One corner stone of the diagnostic process is the dialogue between the family physician (FP) and the patient. Here, the patients are encouraged to tell their stories in their own words [5, 12]. A diagnosis is established based on the symptoms as well as their duration and severity. However, several studies showed that FPs fail to establish correct diagnoses for half of the patients with depression or anxiety disorders [13, 14]. Therefore, a structured interview as a part of the consultation has been proposed as a way of improving diagnostics in primary care [15].

Structured interviews comprise modules for psychiatric disorders, with questions that capture criteria from the two classification systems, Diagnostic and Statistical Manual of Mental Disorders (DSM) and International Statistical Classification of Diseases and Related Health Problems (ICD). The Mini International Neuropsychiatric Interview (MINI) has high diagnostic accuracy for depressive [16] and anxiety disorders [17]. Thus, the MINI might have the potential to improve the diagnostic procedure in primary care for patients with psychiatric problems.

However, indications exist that the MINI seldom is used among FPs in primary care. A questionnaire survey to a large random sample of Swedish FPs found that less than 3 % of the respondents used the MINI [16]. The time required for assessment, usually 20–25 min, was one main obstacle [15, 18]. As the use and acceptance of the MINI in primary care is sparsely studied [18], it is unclear what other barriers there are for the use of the MINI. From studies investigating the use of similar tools, such as short patient questionnaires for depression, other barriers can be identified. Researchers found that FPs were reluctant to use the questionnaire and that the FPs preferred to rely on their clinical experience [19–24]. The FPs perceived that the use of standardized instruments did not fit their professional role and that their consultations were disturbed when questionnaires were introduced.

With these barriers in mind, an alternative could be that the FPs refer patients for assessment with the MINI by other care professionals and thereafter integrate the results in their diagnosis and treatment decisions. Therapists, who are trained to use psychological instruments, might be an option for such a task shift. The aim of the present article therefore was to explore whether such an inter-professional diagnostic process would be feasible for FPs, patients and therapists in primary care, and to identify barriers to and enablers of the process by using the COM-B model [25]. COM-B takes both individual and organizational factors into account, and it has a clear behaviour perspective [25]. According to the framework, the behaviour (B) depends on three components: physical and psychological capabilities (C), physical and social opportunities (O), and reflective and automated motivation (M). The components can exert a direct or indirect effect on the behaviour, and the components may interact with one another.

## Methods

### Design and setting

In 2013 the Stockholm County Council healthcare administration decided to support the implementation of the MINI in primary care. A naturalistic pilot study that investigated the feasibility of the MINI and of a referral to a therapist for the assessment was undertaken. It should serve as guidance for the implementation in accordance with the Medical Research Council guidelines [26]. The study was carried out in three primary health care centers (PHCC) in Stockholm County between February 2014 and March 2015. Findings pertaining to perceptions about the MINI itself and its use have been reported elsewhere [27]. Here we report the results that are related to the diagnostic process.

In Sweden, primary care receives funding from the county councils, which either manage PHCCs on their own or assign them to private healthcare providers. The PHCCs employ a range of professions, e.g. FPs, either certified or under specialist training, district nurses, physiotherapists and, increasingly, psychosocial teams with psychologists and social medical workers. PHCCs are the first level of care for psychiatry. Patients are assessed by an FP. If patients are considered to have a mental problem requiring treatment, the FP either initiates drug treatment or refers them to psychosocial teams or to a psychiatrist.

### Participants and process

Seven PHCCs were approached, based on personal contacts, and three agreed to participate. One PHCC was excluded from this analysis, as only the FPs conducted the MINI. The two remaining PHCCs are described in Table 1.

**Table 1:** Characteristics of participating primary health care centers (PHCC)

PHCC ID	Location, listed patients (n); CNI^a^, proportion born abroad	Ownership	Number employed FPs	PHCC experience with the MINI or similar interviews	
1	Suburb, *n* = 18,000 CNI = 1.26 28% born abroad [28]	County	15	None	
2	Suburb, *n* = 21,000 CNI = 0.93 28% born abroad	Private, new owners and new manager during the study time	14	FPs were trained and encouraged to use the MINI	

^a^CNI = Care Need Index [29] a measure of psychosocial burden, where higher values indicate larger problems; average CNI = 1.0; FP = Family practitioner

Seventeen FPs participated in the focus group discussions (nine from PHCC1 and eight from PHCC2, 14 females in total). They had varying levels of work experience ranging from being under specialist training to having more than 20 years of experience as a certified FP. The distribution of experiences was similar at the two centers and considered to be representative of skills and working methods in Swedish primary care.

Adults with symptoms that were suggestive of depression or anxiety disorders were eligible for assessment with the MINI (for more details see [27]). Patients who needed immediate treatment or had cognitive or language problems were excluded. In total, 110 patients were referred to a therapist and consented to participate in the full pilot study, including a question about the satisfaction with the referral. Of these, 40 signed up tentatively for a research interview. Nine women were not contacted in order to maximize variation. Finally, 22 patients consented and participated in the interviews (Table 2).

**Table 2:** Characteristics of patients who participated in the interviews (*n* = 22)

Characteristics	PCC1	PCC2
Total number of patients	14	8
Women	10	6
Age distribution		
< 25 years	4	1
25–60 years	7	5
> 60 years	3	2
Occupation		
Studies	1	1
Employment	10	5
Retired or unemployed	3	2

Use of the MINI as well as referral to a therapist for MINI assessment was voluntary for the FPs. The diagnostic process in the study plan comprised three steps, listed in Table 3, column 1. The first step was a decision to use the MINI. The second step was a decision to refer to a therapist for the MINI, instead of the FP doing it. The third step was feedback of the results from the therapist, to be used as part of the diagnosis made by the FP. For the feedback, a face-to face communication was recommended. In the present article we are focusing on steps two and three.

**Table 3:** The three step inter-professional diagnostic process for patients with suspected depression or anxiety disorders in primary care

Description of the stages in the intended process	Content of the actual process, PCC1	Content of the actual process, PCC2
The patient meets an FP. 1. The FP decides whether there is a need for the MINI in the diagnostic process	As the intended process	As the intended process
2. The FP considers whether to refer for a MINI assessment by a therapist. The FP suggests referral for eligible patients and explains the reason for the referral.	As the intended process	As the intended process with an assumption that the therapist could treat if indicated
Referral to the therapist according to standard routines	As the intended process	As the intended process
The patient visits the therapist for the MINI assessment	As the intended process. The therapist communicated the results to the patients and informed that the FPwould make the treatment decision.	The therapist assessed the patient with MINI and other tests. The therapist discussed and agreed with the patient on therapy.
3. Feedback of the results of the assessment from the therapist to the FP	As the intended process. The therapist provided feedback both in the patient record system and during a personal meeting.	Usually no feedback from the therapist to the FPs, but information about diagnosis and treatment could be read from the patient record system
The FP decides on treatment.	As the intended process. Sometimes the therapist and the FP agreed on the treatment during the feedback meeting.	The therapist made the decision.

PHCC1 chose to follow the diagnostic process. A medical social worker, certified in psychotherapy, was designated for the referral. The local management decided that 3 h weekly in the therapist’s schedule were assigned for the MINI assessments. At PHCC2, the FPs were used to conduct the MINI assessment themselves. However, two psychologists had been recruited, and the FPs got an opportunity to refer to them. No extra time for MINI assessments was assigned for the FPs or the therapists.

Prior to the study, PHCC1 had an introductory training session that aimed to increase awareness that a correct diagnosis is vital for adequate treatment of mental disorders. This training was not needed at PHCC2. Both PHCCs had a session on the evidence for the MINI to increase the motivation to use it. At the same occasion the study plan and steps two and three was presented and discussed.

### Data collection

Two types of data were collected. All 110 patients were asked to rate their satisfaction with the referral with a Visual Analogue Scale (VAS, 0–100) as last part of a questionnaire. Experiences and perceptions about the referral were gathered from semi-structured interviews with 22 patients and the therapists and focus groups with the FPs. The interviews and focus groups were supported with topic guides (Additional file 1) and were audio-recorded. The interviewees were informed that the study was of interest to the Stockholm County Council healthcare administration. None of the interviewees was known to the interviewer beforehand.

AP interviewed the patients individually, and they chose the dates and locations. The interviews were conducted between 2 weeks and 2 months after the MINI assessment. They lasted approximately 30 min, and the patients were offered vouchers for cinema tickets (value of 12 euros each).

AP interviewed the three therapists at their workplaces after the pilot study. All FPs were invited to focus group discussions [30, 31], two at PHCC1 and one at PHCC2. AP and IK moderated the discussions, ensuring that all participants were involved. The interviews and the focus groups lasted for around 45 min. The only compensation was a light meal in connection with the meeting. A preliminary analysis of the barriers to and enablers of the diagnostic process was presented at a meeting with FPs and with the therapist at PHCC1 in Autumn 2016. The participants found the results to be in accordance with their experiences.

### Data analysis

The VAS scores for satisfaction with the referral were estimated with a ruler and the medians and interquartile range (IQR) were calculated. Ten patients from PHCC2 did not rate the referral.

The interviews were transcribed verbatim. Analyses of the interviews and focus groups were based on qualitative content analysis [32–34]. AP coded the transcripts, and SM verified the codes. SM and AP independently defined subcategories and categories, and they were agreed upon by consensus.

Two analyses were performed. The first served to explore the experiences of the diagnostic process, with the process steps serving as main categories. The second was aimed at identifying factors that influenced whether the FP referred. The COM-B components were used as main categories. For both analyses, an inductive content analysis was performed for each main category. Finally, categories across the participant groups were created. The whole process was continuously carried out forwards and backwards, considering other possibilities, and involved all authors.

## Results

### Adoption of the process and patient satisfaction with it

At PHCC1, nine out of the 15 FPs referred patients (average of seven patients, range one to 17 per FP) according to the study plan. After feedback of the results, the FPs made their diagnosis and initiated treatment. At PHCC2, a third diagnostic process had developed (See Table 3, column 3). The FPs had discontinued their previous use of the MINI.. Patients with complex problems were referred to the therapists for more comprehensive examinations including the MINI. The therapists then, made the final diagnosis and initiated psychological treatment. There was no further involvement from the FPs, except when the therapist judged that referral to psychiatry was warranted (Table 3, column 3).

According the answers to the question “How satisfied are you of being interviewed by a therapist as part of clarifying your problems?” the patients appreciated the referrals to therapists. The median satisfaction was 92 (ICR 75, 100; *n* = 102 patients, no differences between PHCCs).

Many patients had previous experiences from visiting health care facilities for psychiatric problems. In the interviews, they compared the visits and perceived that the diagnostic process including referral to a therapist was more thorough.

### Enablers of and barriers to the process

Although the aims of the referrals differed between the PHCCs, many associated barriers and enablers were similar. The identified factors that could influence referrals and feedback discussions, respectively, are described in Table 4. Representative quotations for the findings are compiled in Table 5 and referred to in the text.

**Table 4:** Factors influencing an inter-professional diagnostic process for depression and anxiety in primary care

Component in COM-B [25]	Factor influencing referral	Factor influencing a feedback discussion	Respondents
Capabilities, psychological	FPs’ competence in terms of knowledge and skills		FPs, therapists, patients
Opportunities, physical	The priorities of the management		FPs, therapists, patients
	Limited time for FPs’ assessment		FPs, therapists, patients
	Easy access to a therapist		FPs
Opportunities, social	Patient characteristics		FPs
	Process facilitator or change agent available		FPs, therapists,
Motivation, reflective	Beliefs that referral facilitates the work of the FP		FPs, therapists, patients
	Beliefs that collaboration on diagnosis can improve patient management		FPs, therapists, patients
	Beliefs about professional roles		FPs, therapists
Motivation, automatic	Positive experiences facilitate a new habit		FPs, therapists
	Easier to continue working as usual		FPs

*FP* Family Practitioner

**Table 5:** Sample quotations for analysis of factors that influence the referral to a therapist for assessment with the MINI, with COM-B as framework

Influencing factor	Sub-category	Sample quotation
FPs’ competence in terms of knowledge and skills	Knowledge about mental disorders	Q1. One has to consider whether there is a need for the MINI (and referral) or if my own assessment is sufficient (FP 8, PHCC 1)
		Q2. The doctor knows about injuries and the therapist about the soul (Patient 1).
	Communication skills	Q3. I was not really prepared for the visit to the therapist. My FP had only told me that I […] needed to talk to a therapist, which I felt was a good idea. This meant that I did not know that I was about to have an interview and the MINI test. […] So, the therapist was a bit unprepared as well and had to explain what it all was about. However, then we talked about what I needed to discuss with her – before the interview. I felt that this was doing it backwards, we should have made the interview first and talked afterwards (Patient 16).
	Consultation skills	Q4. Doctors are doctors after all, they kind of, how shall I express it, take over, I am not in charge anymore. (Patient 9).
The priorities of the management	Lack of supportive routines	Q5. …and there are no fora (for discussions). If you need to contact someone, you have to go and knock on the door when that person is not occupied and you yourself is not occupied. This is very tricky. The alternative is the lunch room… which is well, so-so, you don’t want to do that for all your patients (therapist, PHCC2).
		Q6. The administrative extra tasks have to be taken into consideration e.g. to have a specific time for discussions with the therapist instead of the therapist coming to me in the staff room or in the corridor (FP, PHCC1).
Limited time for FPs’ assessment		Q7. I have had many new patients with short time slots. They present pain and symptoms in different places and you get the impression that depression, anxiety or something similar is behind it all. Then it is important to have this resource (the therapist) since you cannot do that assessment in 15 min (FP 3, PHCC1).
		Q8. Stressed doctors have difficulties to sense who you are and to give the adequate questions about your health (Patient 3).
Easy access to a therapist		Q9. I have reasoned that as the waiting time to the therapist is so short, I have referred them (the patients) before initiation of treatment… and then I have perceived that my depression diagnosis is more robust (FP 9 PHCC1).
Patient characteristics		Q10. You and the patient have to agree on what to do. Especially depressive and psychiatric diagnoses, they are extra sensitive. Had this been a project concerning diabetes, it goes without saying, you would refer the patient (FP 6, PHCC 1).
Process facilitator or change agent available		Q11. The therapist chased us in order to catch us for the feedback. I guess it has been heavy for her (FP 9, PHCC1).
		Q12. Initially, it took time to fill the time slot (assigned for assessment by the MINI) so I sent out reminders in the patient record system in order to get it moving (therapist, PHCC1).
Beliefs that referral facilitates the work of the FP	Saving time and effort	Q13. So, with the therapist assessment, the table was set when the patients came back. It is amazing! The patient was nearly fully examined. So, it saved time for me (FP 4, PHCC1).
		Q14. It is harder for the doctor to arrive at a diagnosis without cooperation with a therapist (patient 1).
	Gives additional information	Q15. …a different climate is needed, kind of, in order for those questions to surface. If you have an hour at your disposal, in an undisturbed context and normal clothing (as is the case for the therapist), the conditions for the therapist are so much better than ours (FP 4, PHCC1).
Beliefs that collaboration on diagnosis can improve patient management		Q16. It is a big advantage that there is someone with whom you can discuss the patient (FP 7, PHCC1).
		Q17. Patients often refer to conversations with the FP and want me to do something. This could be prevented if the FP and I talk and agree on a treatment plan (therapist, PHCC2).
		Q18. I think that you need to meet with a therapist as well as a doctor if you don’t feel well. That the diagnosis and examination are made from both a doctor- and a therapist perspective in order to capture all parts. That the doctor does not label you as being sick “OK take some vitamin-D because you seem to be a bit moody” without the input from someone who is more competent concerning such matters (Patient 18).
Beliefs about professional roles		Q19. But it is something that you feel when you ask the questions (of the MINI) so it becomes an integrated assessment (FP 2, PHCC2).
		Q20. What I do NOT want to see, is that patients are sent to us for an assessment only. An assessment means to create an alliance (between the therapist and the patient) and start planning how patient and therapist can work together, during a treatment. (therapist, PHCC2).
Positive experiences facilitate a new habit		Q21. I began to refer patients that did not fulfil the criteria of the MINI-study as well (FP 3, PHCC1).
		Q22. … and when I have done it once, the doctors understand that they can use me again. So, then they have referred more patients and I have done many more assessments than those included in the study (therapist, PHCC1).
Easier to continue working as usual		Q23. …many that comes to us for help already have a diagnosis from somewhere else. Then it is easy to accept their diagnosis and continue with treatment. Then there is not much of a diagnostic process, although that does not mean that the original diagnosis is the correct one (FP 2, PHCC2).
		Q24. I don’t think that it is self-evident when you see the patient that this person should be referred (for the MINI in a diagnostic process) – it just keeps rolling (FP 8, PHCC1).
The patient perspective		
Most patients accept meeting FPs first		Q25. If I had the opportunity to choose I had visited the doctor first, after all. Because it feels as if the can place you right […], as I said before, I came for stomach problems and did not think at all that I suffered from depression and anxiety (Patient 11).
		Q26. But I can think that it would be good if the doctor could perform the test (MINI) as fast as possible. And if the doctor considers that you suffer from something like --- or if the test suggests that you suffer from something …then the doctor refers you to a therapist (Patient 19).
Importance of personality and knowledge		Q27. It is hard to say who should conduct the test because it depends on background and experience. Maybe the title does not matter much. But the one conducting the test must feel comfortable and understand it. It is not enough to just present the questions and then just fill in “yes” or “no”. Patients might get upset when certain questions are presented, and difficult emotions might come up. So, you have to know how to handle such reactions (Patient 23)

#### Capabilities, psychological

##### FPs’ competence in terms of knowledge and skills

Most FPs believed that they had sufficient competence in psychiatry, especially regarding depression, to make adequate clinical judgements without the MINI. However, they perceived that the MINI could offer extra help with complicated patients, which usually were referred to therapists (Q1, Table 5). Some FPs expressed that their diagnostic knowledge and skills were insufficient due to too little training or experience. These FPs referred patients more broadly, including those with less complicated situations.

The patients often expressed that FPs had insufficient competence in psychiatry. Some of them anticipated that psychiatry was not a part of the FPs’ education. Therefore, according to them, psychiatry was not the business of the FP but for therapists with their education in psychology. Referral was thus seen as natural from the patients’ perspectives (Q2, Table 5).

Furthermore, FPs’ communication of the purpose of the MINI was described as a skill that could influence the patients’ motivation to be referred. Both patients and therapists provided examples where lacking or unclear information from the FP had made the patient feel unsafe or disinterested in the referral (Q3, Table 5) For patients, the consultation style could be problematic. The FP took over the agenda and did not give the patients the opportunity to talk as they had wanted to (Q4, Table 5). Other patients felt that the FPs concentrated on somatic problems instead of trying to find out what was really distressing them. In addition, at PHCC1, the competence of the FP influenced the feedback of the MINI results from the therapist to the FP. The therapist felt that a discussion was especially important for inexperienced FPs.

#### Opportunities, physical

##### The priorities of the management

The priorities of the management of the two PHCCs differed and influenced the process. At PHCC1, the manager encouraged the study and the referral process. She/he was interested in the results and in whether these results would suggest a decision to change routines. The therapist was given time for the assessment, which resulted in easy access to the therapist. At PHCC2, the management at that time introduced a new patient record system, which was given high priority. The FPs got less time to assess complicated psychological symptomatology, which contributed to increased referrals.

*‘Initially, I wanted to conduct the MINI myself and make my own judgements. Now, I have less time and closer to the therapists, so I feel that we could at least share the burden’* (FP 3, PHCC2).

Feedback discussions of the MINI results were hampered by a lack of supportive routines as expressed by FPs and therapists. At PHCC1, the FPs had no time allocated for feedback discussions, which annoyed them. At PHCC2, no policies were in place concerning communication between FPs and therapists. Thus, discussions were few and ad hoc. For both PHCCs, communication tended to take place in the lunch room or during short breaks between patients, which was seen as not acceptable (Q5, Q6, Table 5).

##### Limited time for FPs’ assessment

All respondent groups perceived that the FPs’ consultation times were insufficient for dealing with undetermined psychological symptoms (Q7, Table 5). This was even more problematic when psychological symptoms were not the reasons given for a visit. As expressed by an FP:

*‘I often experience that patients test us. They visit for something else, like acne, but you feel that they are not focused on the problem. They want to see if they can trust you and then, at the end of the consultation, they bring up that they have felt moody for a long time’* (FP 1, PHCC2).

The patients experienced that short consultations led to frustrated FPs who already had the next patient in mind and thus did not listen to them thoroughly (Q8, Table 5).

##### Easy access to a therapist

Short waiting times for a visit with a therapist supported referral at both PHCCs (Q9, Table 5). The FPs emphasised that short waiting times were essential for avoiding the delay of treatment.

#### Opportunities, social

##### Patient characteristics can be a barrier

The FPs perceived that some patients were not keen on being referred for a MINI assessment (Q10, Table 5) Some patients did not believe that they had a mental problem, others with longstanding problems did not want an extra visit to the PHCC. Such patients were not referred to the therapist at PHCC1, as the FPs did not want to persuade patients.

##### Process facilitator or change agent available

At PHCC1, the therapist spontaneously assumed the responsibility of acting as a facilitator. Initially, the FPs forgot to refer patients, and the therapist sent reminders. The FPs appreciated this and increased their referrals. The therapist also took every opportunity to share the MINI results with the FP orally, as recommended in the guidance for the study (Q11, Q12, Table 5).

#### Motivation, reflective

##### Referral facilitates the work of the FP

According to all respondent groups, the diagnostic process saved the FPs’ time and effort (Q13, Q14, Table 5) The FPs compared the process with referrals for somatic problems, e.g. spirometry, that specialised asthma nurses performed. FPs at PHCC1 expressed that psychiatric problems should be handled in the same way and appreciated the diagnostic process.

Another advantage was that the therapists could offer the patients a more comforting atmosphere and more time for reflection and building confidence than what was possible during an FP consultation, according to the respondent groups at PHCC1. As a result, the patient could provide more well-grounded information, which the FPs at PHCC1 saw as a benefit (Q15, Table 5).

##### Collaboration on diagnosis can improve patient management

All respondent groups agreed that an inter-professional diagnostic process could provide benefits to the patient (Q16, Q17, Table 5). The patients expressed that a major benefit was that the assessment was made from both a medical and a psychological perspective (Q18, Table 5).

At PHCC1, such cooperation was established to a varying degree. The therapist presented the results, and often the FP and the therapist discussed how to proceed with the patient. This included treatment options as well as whether the patient needed referral to psychiatry. However, some FPs preferred to ponder the results and did not involve the therapist in the treatment choice.

At PHCC2, minimal collaboration took place between FPs and therapists, except for when a therapist judged that a patient needed referral to psychiatry. In these cases, the therapist prepared the referral documents. Often, the therapist said that the FP just signed them without further discussion. However, the therapists and the FPs noted that feedback on decisions could be valuable, e.g. in the form of a short meeting. According to them, patients could feel confused by the perceived different messages from the FP and the therapist. Some patients confirmed this as well.

##### Beliefs about professional roles

Beliefs about professional roles affected the motivation for referral and feedback discussions. At PHCC1, the inter-professional process did not interfere with existing roles. The FP maintained the patient responsibility, including decisions to refer patients for CBT. A discussion on results and actions to be taken was seen as rewarding among both FPs and therapist.

The therapist perceived increased competence with the new task. Furthermore, becoming more familiar with the MINI, the therapist took her own initiatives, using the MINI for non-assessed patients where the referral form seemed vague and then communicating the results to the FP.

At PHCC2, neither the FPs nor the therapists appreciated the concept of referral for MINI assessment only, albeit for different reasons. The FPs trusted their own assessment skills and felt that they would miss important non-verbal information required for their own diagnostic work if a therapist made the assessment (Q19, Table 5). For the therapists, the diagnostic process was seen as part of creating an alliance and a treatment plan with the patient and thus as an important part of their work. Assisting FPs in their diagnoses was not seen as part of their duties, and furthermore, they did not trust the FPs’ ability to interpret the results from the MINI (Q20, Table 5).

#### Motivation, automatic

##### Positive experiences facilitate a new habit

Some FPs at PHCC1, as told by the FPs and the therapist, changed their habits. They had positive experiences with their first patients and increased their referrals, widening to patients who were outside the scope of this study, e.g. patients with suspected eating disorders (Q21, Q22, Table 5). However, others lost interest in the referral when some patients declined the offered assessment.

##### Easier to continue working as usual

The urge to continue working according to established routines was a strong factor against referral, according to the FPs (Q23, Q24, Table 5). It did not matter that they were aware that referrals were useful.

The FPs also expressed that, for patients who already had been diagnosed elsewhere, it was easier to accept the diagnoses and initiate treatment, even though these diagnoses might not be the correct ones.

### The patient perspective

Patients expressed perceptions that did not directly influence the referral process but were relevant in understanding the perceptions of this process. These concerned their first visits to FPs and the profession they considered were best suited for conducting MINI assessments.

#### Most patients accept meeting FPs first

Many patients were satisfied with meeting FPs first and then being referred to therapists for assessment (Q25, Q26, Table 5). If they were dissatisfied, it most often was related to mistrust, of either the FPs or the therapists, rather than to the referral procedure. Some patients, though, found the FP visits to be unnecessary. They perceived that they already knew their problems and wanted psychological treatment immediately. They saw the FP visits as a formality without any clear benefit. Others wanted to shorten the time to treatment and suggested that the FP could conduct the MINI during the visit rather than referring the patient to a therapist.

#### Importance of personality and knowledge

Even if a patient appreciated a referral to a therapist, it was not self-evident that the therapist was the most appropriate for conducting the MINI. The profession itself was not regarded as crucial. The personality, knowledge and experience of the person conducting the MINI interview played a more important role (Q27, Table 5). The interviewer should, for example, be familiar with any unwanted emotions during the MINI and know how to handle them. Some patients were confident that their FPs could conduct MINIs effectively. Others were indifferent and perceived that it did not matter whether an FP or a therapist conducted the MINI; they would accept both.

## Discussion

We found that referral to a therapist for MINI assessment with feedback to the FP can be feasible in primary care. This would be an approach closer to the established standards for the diagnosis of mental disorders [35]. We also found that referrals to therapists for MINI assessments were influenced by the FPs’ knowledge in psychiatry and their beliefs that the process saved time and improved patient care. Furthermore, the priorities of the management and established habits influenced whether FPs referred patients to therapists. The second part of the inter-professional diagnostic process, i.e. a feedback discussion of the MINI results between the FPs and therapists, was influenced by existing routines, the division of tasks and views on professional roles.

The idea was to build on the routines used for somatic diseases, where FPs refer to other professionals for more comprehensive examinations but retains the patient responsibility including final diagnosis and decision on treatment. To our knowledge, referral to a therapist was not studied earlier in primary care settings. However, two other studies found that referral to a nurse for assessment with structured interviews was well accepted among FPs in primary care [36, 37]. However, these studies focused on time saving only and did not include feedback discussions.

The adoption of the diagnostic process could be hampered by beliefs from FPs and therapists on responsibilities and professional roles, which was illustrated in the present study.

At PHCC2, the FPs did not consider the reasons for referral to assessment only convincing enough. The FPs perceived that non-verbal information was lost if the assessment with the MINI was made by someone else, and that they trusted their own competence. The therapists stressed that diagnosis was part of the treatment and that they doubted that FPs could interpret the results. However, due to lack of time the FPs handed over the diagnosis of complicated patients to the therapists with the underlying assumption that the therapists would also initiate psychotherapy if appropriate. This task division would be more in line with models such as Collaborative Care (Gunn 2006 [38]) or IAPT (Clarke 2011 [39]).

Although the diagnostic processes were partly different at the PHCCs, the identified barriers to and enablers of the process were mostly similar. The COM-B analysis revealed that 11 main factors influenced the diagnostic process. First, the short consultation times the FPs had with patients and their beliefs that referrals relieved their workloads were driving forces for the FPs to refer patients to the therapists. This is in contrast to a prior study [40] where FPs and nurses expressed that opportunities for learning from each other and exchanging ideas, rather than decreased workloads, enabled their inter-professional work. In our study, the time might have been a crucial factor because FPs in primary care have high demands with regards to meeting many patients per day to meet the reimbursement targets.

The FPs’ knowledge and skills in psychiatry were identified as another determinant of practice and mainly operated as an enabler. FPs with less experience saw referrals as positive opportunities for a broad range of patients. However, most FPs perceived themselves as having good capabilities in the diagnosis of especially depression, in line with the current literature [12]. For them, referral was chosen for patients with more complex problems, which was reported in another study [41]. Easy access and working relationships with a therapist were essential for the FPs, which has also been seen in other studies [41, 42].

The second part of the process was the feedback discussion of the MINI results, which required collaboration between the FPs and the therapists. The patients expressed that teamwork between the professionals was essential for the diagnosis of mental problems. However, at the outset, no teamwork occurred, as the FPs and therapists worked independently from each other. The success of the feedback part turned out to depend on professional expectations regarding work content. At PHCC1, feedback discussions acted as enablers, as they were seen as rewarding for both FPs and therapists. Prior to the study, the duties of the therapist had been restricted to treatments after referral from a FP. The diagnostic process meant a new competence-increasing task for the therapist. However, the roles were maintained, which the therapist accepted. At PHCC2, the therapists expected to have full responsibility for diagnosis and treatment, and they had strong objections against a process where the FPs made the final diagnoses. A feedback on the diagnosis and treatment was not seen as necessary. The FPs accepted this, although they had wished for better communication. This behaviour was seen in another study, where the implementation of inter-professional work was impeded by a mutual wish to ‘stay apart’ [42]. Studies have mostly shown that perceptions about professional roles and identities are common barriers to inter-professional work [42–44].

Another barrier was that routines for supporting a feedback discussion were not in place. This was illustrated by the difficulties with accomplishing the feedback at PHCC1. Although the management assigned time for the therapist, no time was allocated for the FPs for the feedback discussions. This finding is in accordance with the results from several other studies [42–45]. Finally, an enabler of the whole process was the availability of a process facilitator or change agent. At PHCC1, the therapist spontaneously took the role. The therapist was seen as central to the success of the referral process by sending reminders and ensuring that the FPs had detailed feedback. The importance of a facilitator or change agent has been shown in many studies, as summarized in [46].

The identified barriers and enablers are similar to those found for Collaborative Care in two systematic reviews [47, 48]. They found that buy-in was an important determinant for implementation. Lack of time, competing priorities, uncomfortable with setting a diagnosis and treat mental problems decreased buy-in. Perceptions that Collaborative Care gave benefits for the patient increased buy-in. Face-to face meetings were critical for collaboration between professions, according to the reviews. However, the meetings increased work load which was seen as a problem.

An important issue that is often overlooked is the patient perspective [43]. We found that patients were very satisfied with the referral. However, our findings did not suggest that positive patient attitudes motivated the FPs to refer. On the contrary, the experiences of meeting some unwilling patients were arguments for not referring. Whether patients’ perspectives influence the implementation of new work methods has been studied very little [49]. In our study, patients had many thoughts about professional roles and about who should be responsible for the diagnoses, which can be useful information. Although referrals for MINIs were appreciated, some patients preferred that FPs conduct the MINIs. Others wanted to meet with a therapist directly.

### Strengths and limitations

As the process included a modification of professional roles, the study contributes to the literature on environmental restructuring, where the evidence is currently limited [50].

A strength of this study is that it took the perspectives of patients as well as FPs and therapists into account. This provided a finer-grained view of factors that can impact the inter-professional process, rather than simply listening to the FPs only. Another strength is that the analysis was based on an established behaviour model, COM-B. As the model is increasingly used (see e.g. [51–53]) it will facilitate future comparison with other studies that have used the model.

The study had several limitations. The main limitation relates to the selection of PHCCs. Participation required that the managers be willing to invest in time for the assessment and for data collection. This resulted in the inclusion of only two PHCCs, where one had several positive buy-in factors influencing the process whereas the second had not. Thus, the study only indicates that given positive prerequisites for buy-in the intended process may work. A second issue relates to the completeness of the data collection. All FPs were invited to the focus groups, but only around half of them participated. Most of these FPs had referred patients. Thus, it is possible that more barriers against the use of referrals existed than those we captured.

Finally, it was not possible to gather information about the number of patients that fulfilled eligibility criteria but were not referred or the reasons behind not referring, which had been interesting.

## Conclusions

An inter-professional diagnostic process where FPs refer patients to a therapist for assessment and the results are fed back to the FPs can be feasible. Feasibility depends on access to a therapist, the perceptions of roles and competences among FPs and therapists, and strategies for supporting teamwork.

## Additional file

Additional file 1: Topic guide for the interviews (PDF 165 kb). (DOCX 18 kb)

## Data Availability

Owing to confidentiality agreements with the participants, the interview transcripts are not publicly available. Anonymized transcripts in Swedish are available upon request.

## Abbreviations

CBT: Cognitive behavior therapy
COM-B model: Capability, Opportunity, Motivation – Behavior model
FP: Family practitioner
IQR: Interquartile range
MINI: Mini International Neuropsychiatric Interview
PHCC: Primary Health Care Center
VAS: Visual Analog Scale
