# Supplementary material for: Feasibility of referral to a therapist for assessment of psychiatric problems in primary care – an interview study
Source: BMC Fam Pract. 2019 Aug 19;20:117. doi: 10.1186/s12875-019-1007-7 (PMC6700983; doi:10.1186/s12875-019-1007-7)
Supplement: Supplementary file 1 — Topic guide for the interviews (PDF 165 kb). (DOCX 18 kb) [file 12875_2019_1007_MOESM1_ESM.docx]

**Additional file 1. Topic guides for interviews and focus group discussions***

**Focus group discussions with FPs**

What experiences have you had from referral to therapists for MINI assessment?

- *To what extent did you refer patients to the therapist and what types of patients did you refer?*
- *What referral routines did you use to refer to the therapist?*
- *How did you get feedback? What are your experiences of the feedback?*
- *How was the division of responsibilities between you and the therapist?*
- *Did you encounter any problems in the referral process?*

What advantages and disadvantages did you perceive with the referral for MINI assessment?

**Interviews with therapists**

Which routines for referral to you, for MINI assessment, have been used and what are your experiences of them?

How did you feedback results from the MINI assessment to the FP? What are your experiences from the feedback?

How do you cooperate with FPs?

- *What are your perceptions about the process where FPs refer patients for MINI assessment to you?*
- *How would you like to see the responsibilities regarding diagnosis of mental disorders shared between you and the FPs?*

**Interviews with patients**

Please tell me, without mentioning any diagnoses, why you visited the PHCC.

- *Have you visited health care facilities for similar problems before this visit?*

What happened when you first met with the FP?

- *Why do you think the FP referred you to a therapist?*
- *How did the referral to a therapist for assessment influence your confidence in your FP?*
- *How did the referral to a therapist influence your relation to your FP?*

What happened during the visit to the therapist?

How did you experience the visit?

Did you meet your FP again after the visit to the therapist?

What happened when you met??

*If the patient met the FP:*

- *Did the FP refer to the answers from the interview done by the therapist? Can you tell me more about that?*

What advantages could you see with a referral to a therapist?

What disadvantages could you see with referral to a therapist?

If you were to make a recommendation on procedures for diagnosing mental disorders in primary care, what would you recommend? Why?

*Detailing that some alternatives were that only the FP was involved, that patients could meet a therapist directly without seeing an FP or some kind of collaboration between the two professions.*

* probing questions, used when necessary are in italics
